# FINDSITE^LHM^: A Threading-Based Approach to Ligand Homology Modeling

**DOI:** 10.1371/journal.pcbi.1000405

**Published:** 2009-06-05

**Authors:** Michal Brylinski, Jeffrey Skolnick

**Affiliations:** Center for the Study of Systems Biology, School of Biology, Georgia Institute of Technology, Atlanta, Georgia, United States of America; Stanford University, United States of America

## Abstract

Ligand virtual screening is a widely used tool to assist in new pharmaceutical discovery. In practice, virtual screening approaches have a number of limitations, and the development of new methodologies is required. Previously, we showed that remotely related proteins identified by threading often share a common binding site occupied by chemically similar ligands. Here, we demonstrate that across an evolutionarily related, but distant family of proteins, the ligands that bind to the common binding site contain a set of strongly conserved anchor functional groups as well as a variable region that accounts for their binding specificity. Furthermore, the sequence and structure conservation of residues contacting the anchor functional groups is significantly higher than those contacting ligand variable regions. Exploiting these insights, we developed FINDSITE^LHM^ that employs structural information extracted from weakly related proteins to perform rapid ligand docking by homology modeling. In large scale benchmarking, using the predicted anchor-binding mode and the crystal structure of the receptor, FINDSITE^LHM^ outperforms classical docking approaches with an average ligand RMSD from native of ∼2.5 Å. For weakly homologous receptor protein models, using FINDSITE^LHM^, the fraction of recovered binding residues and specific contacts is 0.66 (0.55) and 0.49 (0.38) for highly confident (all) targets, respectively. Finally, in virtual screening for HIV-1 protease inhibitors, using similarity to the ligand anchor region yields significantly improved enrichment factors. Thus, the rather accurate, computationally inexpensive FINDSITE^LHM^ algorithm should be a useful approach to assist in the discovery of novel biopharmaceuticals.

## Author Summary

As an integral part of drug development, high-throughput virtual screening is a widely used tool that could in principle significantly reduce the cost and time to discovery of new pharmaceuticals. In practice, virtual screening algorithms suffer from a number of limitations. The high sensitivity of all-atom ligand docking approaches to the quality of the target receptor structure restricts the selection of drug targets to those for which high-quality X-ray structures are available. Furthermore, the predicted binding affinity is typically strongly correlated with the molecular weight of the ligand, independent of whether or not it really binds. To address these significant problems, we developed FINDSITE^LHM^, a novel threading-based approach that employs structural information extracted from weakly related proteins to perform rapid ligand docking and ranking that is very much in the spirit of homology modeling of protein structures. Particularly for low-quality modeled receptor structures, FINDSITE^LHM^ outperforms classical all-atom ligand docking approaches in terms of the accuracy of ligand binding pose prediction and requires considerably less CPU time. As an attractive alternative to classical molecular docking, FINDSITE^LHM^ offers the possibility of rapid structure-based virtual screening at the proteome level to improve and speed up the discovery of new biopharmaceuticals.

## Introduction

Ligand virtual screen is widely used in rational drug discovery [1],[2]. The first stage of structure-based ligand screening is the prediction of the binding mode adopted by the small molecule complexed to its target receptor protein; a variety of algorithms have been developed to achieve this goal [3],[4]. The next step is to estimate the relative binding affinity of the docked ligands [5],[6]. Of course, it is not sufficient that a given ligand binds favorably to given protein; rather, to minimize side effects, it must also bind selectively. Classical molecular docking has been used to address both goals. However, only is it computationally expensive, but there are significant issues associated with ligand ranking [5],[7]. Thus, fast and accurate methods for both binding pose prediction and ligand ranking need to be developed.

With the rapid increase in the number of experimentally solved protein structures, protein homology modeling has become a powerful tool in modern structural biology [8],[9]. Comparative modeling methods identify homologous protein structures and use them as structural templates to model the target protein of unknown tertiary structure. Using a high sequence identity template with a clear evolutionary relationship to the target, the modeled target structure can have a root-mean-square-deviation, RMSD, from the native structure <2 Å [10]. In the “twilight zone” of sequence identity [11], structural information extracted from weakly homologous structure templates identified by threading is sufficient to provide approximately correct 3D models for a significant fraction of protein targets [12],[13]. In contrast to protein structure prediction, information from related 3D structures is rarely used in the large-scale modeling of protein-ligand complexes.

One example of an approach that employs such information is CORES, an automated method for building three-dimensional protein-ligand complexes [14]. CORES directly utilizes the conformation and binding pose of key structural elements of the target ligand, termed “molecular frameworks”, found in templates that are closely related to the protein target. Its practical utility was demonstrated on a set of protein kinases in which ligands containing related frameworks were found to bind in the same orientation. A similar approach designed specifically for kinases, kinDOCK, performs ligand comparative docking by using a kinase family profile to align the related kinase-ligand complexes onto the target kinase's structure and then directly transfers the ligand coordinates [15]. KinDOCK typically docks target ligands into the kinase binding pocket within a 2 Å RMSD from the crystal structure. Moreover, an original clustering procedure based on the binding pose similarity was proposed to highlight the structural similarities and differences within a set of multiple X-ray structures complexed with different ligands [16]. Other examples of ligand docking studies that utilize structural information extracted from closely related protein-ligand complexes include the analysis of cathepsin inhibitor specificity [17], the examination of carbohydrate recognition by the viral VP1 protein [18], screening for selective bacterial sirutin inhibitors [19] and the design of small molecule inhibitors of the macrophage migration inhibitory factor [20]. Typically, modeling templates for the target ligands are extracted from 3D structures of small molecules complexed to closely related proteins.

In our previous study, we observed that evolutionarily remotely related proteins identified by threading often share a common ligand-binding site [21]. Both the localization of the binding site and chemical properties of bound ligands are strongly conserved. This forms the basis of the FINDSITE binding site prediction/protein functional inference/ligand screening algorithm [21]. Furthermore, we found that a pocket-specific potential of mean force derived from known protein-ligand complexes identified for a given target sequence by threading is often more specific than generic knowledge-based potentials derived from ligand-protein complexes found in the PDB [22]. This enhanced specificity suggests that the binding mode and protein-ligand interactions in distantly related protein families are conserved during evolution. To confirm this hypothesis, here, we present the results of ligand binding mode analysis of evolutionarily distant proteins identified by state-of-the-art threading methods [23]. The ligands that bind to the common binding site contain a set of strongly conserved anchor functional groups as well as a variable region that imparts specificity to a particular family member. Furthermore, the degree of sequence and structure conservation of residues in contact with the ligand anchor functional groups are higher than those contacting ligand variable regions. Exploiting these observations, we develop FINDSITE^LHM^ (LHM stands for *L*igand *H*omology *M*odeling) that employs structural information extracted from weakly related proteins to perform rapid ligand docking and ranking by homology modeling; we compare its accuracy to classical ligand docking/ranking approaches [4],[22],[24].

## Results

### Binding site prediction by FINDSITE

The protocol followed in this study is a direct extension of FINDSITE [21], a threading-based method for ligand-binding site prediction and functional annotation that detects the conservation of functional sites and their properties in evolutionarily related proteins. For a given target sequence, FINDSITE identifies ligand-bound template structures from a set of distantly homologous proteins (here, we limit ourselves to target proteins having <35% sequence identity to their closest template, but this arbitrary restriction would be removed in real world predictions) recognized by the PROSPECTOR_3 threading approach [23] and superimposes them onto the target's (experimental or predicted) structure using the TM-align structure alignment algorithm [25]. Binding pockets are identified by the spatial clustering of the center of mass of template-bound ligands that are subsequently ranked by the number of binding ligands.

### Ligand anchor substructure identification

**Figure 1:**
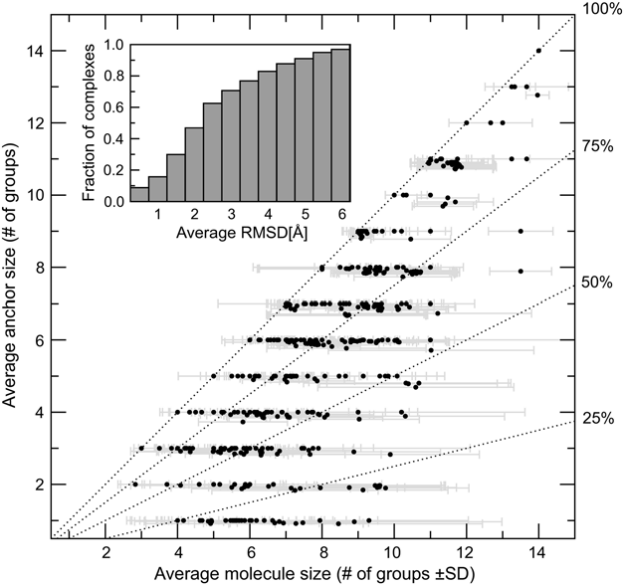
Average molecule size ±SD (one standard deviation) plotted as the function of average anchor size for the largest clusters of similar compounds bound to the top-ranked predicted binding pockets. Dotted lines separate clusters for which different anchor sizes were found (100%, 75%, 50% and 25% of the average ligand molecule respectively). Inset: cumulative distribution of the average pairwise RMSD of the anchor groups upon global superposition of the template proteins.

For each target protein, the template-bound ligands that occupy a top-ranked, predicted binding site are clustered using the SIMCOMP chemical similarity score [26]. The “anchor” substructure is then identified in each cluster as described in Methods. First, we examine the anchor substructure size relative to the average molecule size. Applying the approach to a representative benchmark set of 711 ligand-protein complexes (where the target proteins have pairwise sequence identity to their templates <35%, see Methods), as shown in Figure 1, in most cases, at least 50% of a ligand is comprised of an anchor region whose functional groups are conserved in >90% of the template ligands. Those clusters in which the anchor region is smaller than 50% of the ligand are mostly short oligosaccharides, with a sugar monomer identified as a common substructure. This also explains the high standard deviation in the average ligand molecule size. For some difficult cases, our graph isomorphism analysis didn't provide a sufficient number of atomic equivalences to recognize a common substructure. In contrast, those targets near the diagonal have an anchor equivalent to the average molecule size and represent strongly conserved ligands with little chemical variability; e.g. hemes. In addition, there are targets with a very small number of templates, all having very similar ligands. Nonetheless, for the majority of targets, a well-defined anchor substructure with a co-occurring variable region is detected.

Having identified the anchor substructure, we next investigate the structural conservation of its binding mode. Figure 1 (inset plot) shows the histogram of the average pairwise RMSD among the anchor groups upon global superposition of the template proteins. Note that the properties of the native ligand are not used in any way to identify the anchor region's properties. Clearly, in most cases, the average pairwise RMSD is <2.5 Å.

### Properties of protein binding residues

**Figure 2:**
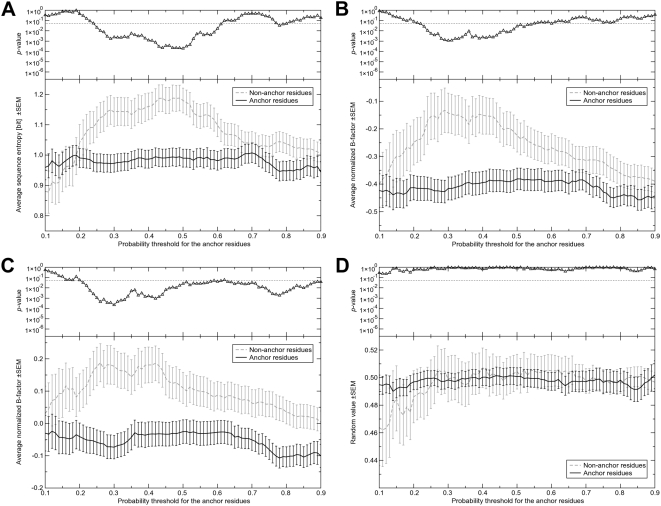
The degree of sequence and structure conservation for the protein's ligand-binding region. (A) Average sequence entropy, average normalized B-factor for (B) the Cα atoms and (C) the side chain heavy atoms as well as (D) a random property assigned to anchor and non-anchor CBRs. The populations of anchor and non-anchor CBRs were determined using different probability thresholds for anchor residues. Top plots show the *p*-value of the *t*-test applied to both populations of CBRs with respect to the property under consideration.

We next examine the properties of the protein's ligand-binding region. Given the chemical conservation of the anchor substructure as well as the strong structural conservation of it's binding mode, for binding residues, one would expect that residues contacting ligand anchor groups are more conserved than average. The degree of sequence and structure conservation was calculated for consensus binding residues (CBRs), defined as residues contacting a ligand in at least 25% of the threading templates. This criterion was previously found to maximize the overlap between predicted and observed binding residues [21] and provides sufficient statistics to calculate the sequence and structural features of binding residues. We used a probability threshold to define anchor/non-anchor CBRs based on the protein-ligand contacts extracted from the threading templates. The probability of a residue to be an anchor residue simply corresponds to the fraction of contacts formed by all residues in the equivalent position in the template structures with anchor functional groups of bound ligands. Differences in the degree of sequence and structure conservation between anchor and non-anchor CBRs were calculated on increasing the probability threshold from 0.1 to 0.9 using Student's t-test for independent samples. Shannon's information entropy is used to measure the sequence variability at a particular position in a target protein (see Methods). Analysis of the sequence entropy revealed a significantly higher sequence conservation of residues in contact with the anchor functional groups than those in contact with ligand variable regions (Figure 2A). Next, we analyzed the structural features of CBRs in terms of the experimental B-factors that reflect local mobility [27] and find that the B-factors of residues in contact with the anchor region of the ligands are significantly lower (Figure 2B and 2C which shows the B-factors of the Cαs and side chain heavy atoms, respectively). The conservation of the anchor-binding pose is consistent with the relatively lower B-factors observed for the residues in spatial proximity to the anchor functional groups. These results differ significantly from random (Figure 2D).

### Ligand binding pose prediction

**Figure 3:**
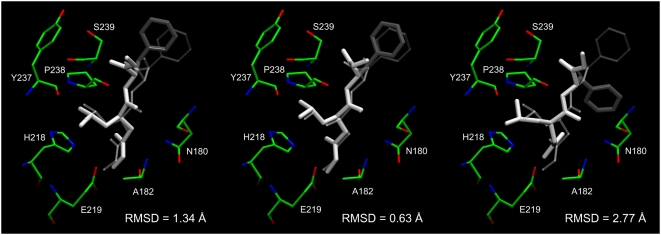
Ligand binding pose prediction for human fibroblast collagenase (PDB-ID: 1hfc). Predicted poses (thick, solid) from FINDSITE^LHM^: (left) superimposed ligand with the anchor portion colored in white, (middle) minimized conformation with Amber and (right) generated by AutoDock are compared to the experimental binding pose of hydroxamate inhibitor (thin, transparent). RMSD values were calculated for heavy atoms. Selected binding residues are shown.

Given that Figure 1 (inset) strongly suggests that the localization of the anchor substructure and its internal conformation is conserved, we developed FINDSITE^LHM^, a very simple, rapid approach for ligand binding pose prediction. Using the consensus-binding mode of the anchor substructure, we align the ligand of interest to the anchor region and then, optionally, minimize the ligand conformation to remove steric clashes. This procedure can be thought of as “ligand docking by homology modeling”. Here, only weakly related template proteins (<35% sequence identity to the target) selected by threading were used to derive the consensus anchor-binding mode. In Table 1, using the crystal structures as the target receptors for ligand docking for the 711 ligand-protein set, the results are compared to three established ligand docking approaches [4],[22],[24] in terms of the heavy atom RMSD from the crystal structure. Target proteins are divided into three subsets with respect to the coverage of the predicted anchor substructure. For the first subset (full coverage) that consists of proteins for which a portion of their target ligands cover at least 90% of the functional groups in the predicted anchor substructure, simple ligand superposition is quite successful and outperforms regular ligand docking approaches. For these cases, using all-atom minimization with Amber [28], the predicted binding mode can be refined to an average RMSD from the crystal structure of ∼2.5 Å. An example of successful refinement is presented for the human fibroblast collagenase in Figure 3, where the final ligand heavy atom RMSD is 0.63 Å. In contrast, the RMSD from AutoDock is 2.77 Å.

**Table 1:** Docking results for the FINDSITE^LHM^ dataset in terms of ligand heavy atom RMSD from the crystal structure.

Docking algorithm	Full coverage*	Partial coverage†	Low coverage‡
Targets§	522	142	47
FINDSITE^LHM^ ¶	2.81±2.15	4.79±2.33	5.08±2.08
FINDSITE^LHM^+minimization∥	2.55±2.28	4.70±2.52	5.03±2.20
AutoDock	3.12±2.61	4.34±2.71	3.88±3.15
Q-Dock	3.26±2.12	4.93±2.35	4.90±2.21
LIGIN	4.70±2.59	4.86±2.59	4.46±2.52
Random	5.85±1.67	5.74±1.58	5.02±1.49

RMSD values are reported for three subsets comprising ligands with different anchor region coverage.

*: Target ligand covers ≥90% of the anchor functional groups.

**†:** Target ligand covers ≥50% and <90% of the anchor groups.

**‡:** Target ligand covers <50% of the anchor groups.

**§:** Number of target proteins.

**¶:** Ligand superposed onto the consensus anchor-binding mode.

**∥:** Superposed conformation minimized with Amber. All results are in Å.

**Figure 4:**
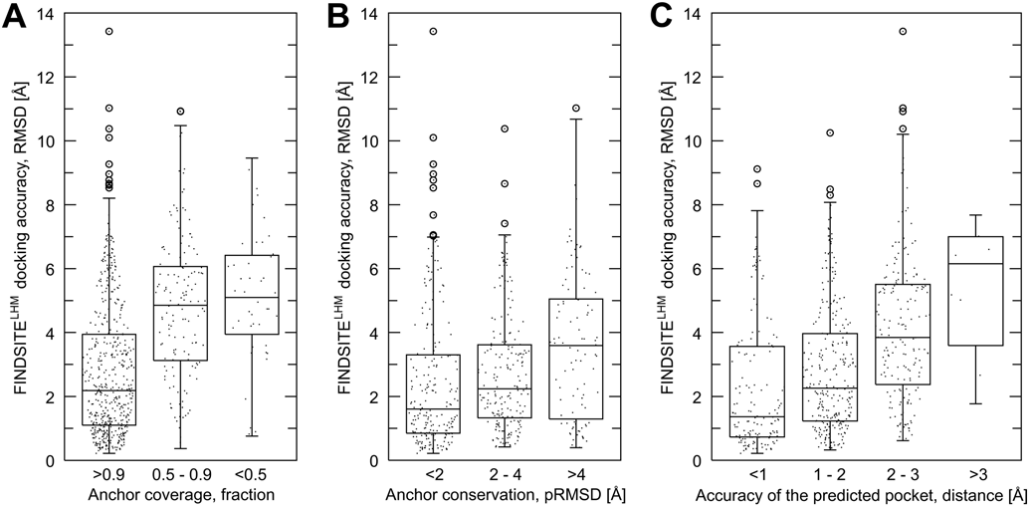
Confidence index for ligand docking by FINDSITE^LHM^. Box and whiskers plots of the relationship between the accuracy FINDSITE^LHM^ in terms of the RMSD from the crystal ligand pose calculated for its heavy atoms and (A) the coverage of the anchor substructure by a target ligand, (B) the structural conservation of anchor binding mode expressed as the average pairwise RMSD (pRMSD) of the anchor functional groups, and (C) correlation between the pocket prediction accuracy by FINDSITE assessed by the distance between the predicted pocket center and the predicted center of mass of the native ligand. Boxes end at the quartiles Q_1_ and Q_3_; a horizontal line in a box is the median. Whiskers point at the farthest points within 1.5 times the interquartile range and circles represent the outliers.

The second subset (partial coverage) comprises target ligands that do not fully cover any of the predicted anchor substructure. Here, the average RMSD of the binding mode predicted by FINDSITE^LHM^ is higher than AutoDock and is comparable to Q-Dock and LIGIN. However, it is still better than random ligand placement. Finally, if none of the predicted anchor substructures are even partially covered by a target ligand (low coverage), the results of docking using FINDSITE^LHM^ are indistinguishable from random. Here, traditional ligand docking approaches, particularly AutoDock, give much better results. In addition to anchor structure coverage, the performance of FINDSITE^LHM^ depends on the overall accuracy of binding pocket prediction and the conservation of the anchor-binding mode; this is discussed in further detail below and presented in Figure 4, see below. Here, we note that using the fraction of the anchor region that is aligned for a given ligand (Figure 4A), or the average pairwise RMSD of the anchor ligand functional groups (Figure 4B), we can predict the expected accuracy of binding pose prediction without knowing the experimental result. Not surprisingly (Figure 4C), when the accuracy of the binding pocket prediction as provided by FINDSITE improves, the accuracy of the ligand pose prediction by FINDSITE^LHM^ also improves.

Weakly homologous protein models frequently have significant structural inaccuracies in side-chain and backbone coordinates and thus, are much more challenging targets for ligand binding pose prediction. The performance of FINDSITE^LHM^, AutoDock, Q-Dock and LIGIN in ligand docking when protein models are used as the target receptors was assessed for the Dolores dataset of 205 proteins [22],[29]; the average Cα RMSD to native of these protein models is 3.7 Å. Table 2 presents ligand docking results using crystal structures as well as weakly homologous protein models in terms of the fraction of recovered binding residues and specific native contacts. Considering the complete dataset and receptor crystal structures, the accuracy of FINDSITE^LHM^ is slightly lower than AutoDock and Q-Dock. This is because the predicted anchor substructure was fully covered (≥90%) by the target ligand only for 62.4% of the receptors; partial (≥50% and <90%) and low (<50%) coverage of the anchor substructure was found for 25.4% and 12.2% of the targets, respectively. This partly reflects the fact that the placement of the ligand variable region has a random component that diminishes the overall accuracy. Consistent with the decrease in ligand RMSD on minimization, the fraction of binding residues and native contacts increases.

**Table 2:** Docking results for the Dolores dataset in terms of the fraction of recovered binding residues and specific native contacts.

Docking algorithm	Binding residues		Native contacts	
	Crystal*	Model†	Crystal*	Model†
Targets‡	205 / 166 / 120	205 / 164 / 117	205 / 166 / 120	205 / 164 / 117
FINDSITE^LHM^ §	0.64 / 0.70 / 0.76	0.55 / 0.61 / 0.66	0.46 / 0.52 / 0.59	0.38 / 0.43 / 0.49
FINDSITE^LHM^+minimization¶	0.67 / 0.73 / 0.79	0.53 / 0.59 / 0.63	0.47 / 0.53 / 0.61	0.28 / 0.32 / 0.35
AutoDock	0.73 / 0.77 / 0.82	0.50 / 0.54 / 0.57	0.52 / 0.57 / 0.64	0.25 / 0.27 / 0.30
Q-Dock	0.77 / 0.81 / 0.85	0.64 / 0.70 / 0.74	0.51 / 0.55 / 0.63	0.39 / 0.45 / 0.50
LIGIN	0.64 / 0.69 / 0.72	0.47 / 0.50 / 0.53	0.39 / 0.42 / 0.46	0.20 / 0.22 / 0.23
Random	0.55 / 0.60 / 0.63	0.50 / 0.54 / 0.57	0.27 / 0.30 / 0.32	0.23 / 0.25 / 0.27
Direct transfer∥		0.77 / 0.78 / 0.78		0.69 / 0.70 / 0.71

Three values (A/B/C) are reported for: (A) all targets, (B) FINDSITE “Easy” targets with at least partial anchor coverage and (C) FINDSITE “Easy” targets with full anchor coverage.

*: Crystal structures.

**†:** protein models used as targets for binding site prediction and ligand docking.

**‡:** Number of target proteins.

**§:** Ligand superimposed onto the consensus anchor-binding mode.

**¶:** Superimposed conformation minimized with Amber.

**∥:** Ligand transferred directly from the crystal structure.

In contrast, for protein models, FINDSITE^LHM^ recovered more binding residues and specific native contacts than both all-atom docking approaches, AutoDock and LIGIN. Considering only the most confident cases for which FINDSITE was likely to predict the binding pocket center with ≤4 Å accuracy (“Easy” targets) and the predicted anchor substructure fully (partially) covered by the target ligand, the fraction of binding residues and specific native contacts recovered by FINDSITE^LHM^ is 0.66 (0.61) and 0.49 (0.43), respectively. However, now the all-atom minimization procedure applied to the binding poses predicted by FINDSITE^LHM^ caused a loss of the specific native contacts. This reflects the fact that structure adjustments are required to remove the repulsive ligand-residue interactions that are not accommodated by simple minimization. Nevertheless, these results represent a significant improvement over traditional all-atom docking against modeled receptor structures. We also note the high sensitivity of all-atom docking approaches to the quality of the receptor structures; for weakly homologous protein models, the performance of AutoDock and LIGIN is no better than random ligand placement into the predicted binding sites. The performance of Q-Dock for protein models was notably higher, since it was explicitly designed to deal with structural inaccuracies in predicted receptor models. Finally, in contrast to classical ligand docking approaches, FINDSITE^LHM^ is computationally less expensive, and typically requires less than a minute of CPU time (see Table S1).

### FINDSITE^LHM^ docking confidence

An interesting question that is very important from the practical point of view, is when should we expect a successful binding mode prediction by using ligand docking by homology modeling? In addition to the coverage of an anchor structure that clearly impacts docking accuracy (Figure 4A), we also investigated the relationship between pocket prediction accuracy, expressed as the distance between the predicted pocket center and the geometric center of the native ligand, the conservation of anchor binding mode in terms of the average pairwise RMSD of the anchor functional groups, and the accuracy of FINDSITE^LHM^ binding mode prediction assessed by the heavy atom RMSD from the crystal ligand pose. As expected, the average accuracy of the binding mode prediction by FINDSITE^LHM^ decreases with decrease in the degree of the conservation of the anchor substructure (Figure 4B). The RMSD of the predicted ligand-binding pose is <2 Å on average for highly conserved anchor substructures whose pairwise RMSD is <2 Å. For moderately conserved anchor substructures with a pairwise RMSD of 2–4 Å, the RMSD of the predicted ligand-binding mode is <3 Å in most cases. Finally, accompanied by weak (>4 Å) structural conservation of an anchor, docking accuracy drops to >3 Å on average. In addition, the drop off in ligand binding pose prediction correlates with the overall accuracy of binding pocket prediction by FINDSITE (Figure 4C). The most accurate ligand binding poses were obtained for precisely detected pockets, where the pocket center was predicted within 2 Å from the geometric center of the native ligand. Considering the structural conservation of the derived anchor substructure, its coverage by a target ligand and the FINDSITE confidence index for pocket detection [21], (all properties which can be calculated *without* knowledge of the native binding pose), one can roughly estimate the quality of the performance of FINDSITE^LHM^ in ligand binding pose prediction.

### Anchor region identification and analysis

**Figure 5:**
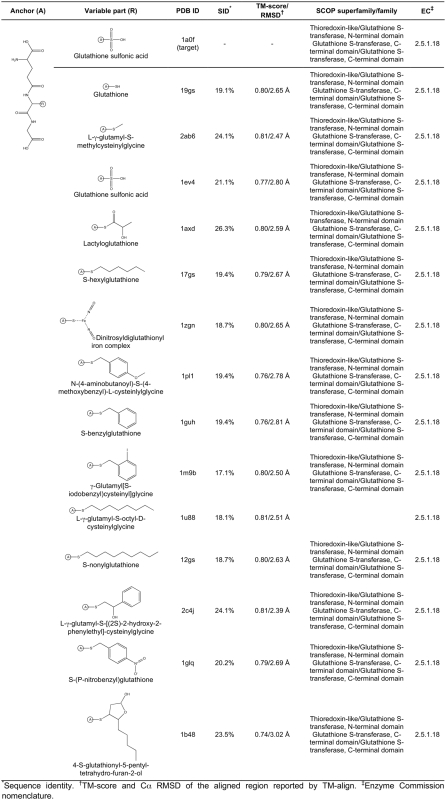
Ligand anchor identification for glutathione S-transferase from *E. coli* (PDB-ID: 1a0f). Common anchor substructure (A) identified from weakly homologous threading templates as well as different variable groups (R) found in ligands complexed with the template proteins are presented.

The results of the application of FINDSITE^LHM^ to glutathione S-transferase (PDB-ID: 1a0f), MTA phosphorylase (PDB-ID: 1sd2) and lysine aminotransferase (PDB-ID: 2cjd) are presented. Figures 5–10 present the common ligand anchor substructures/variable groups identified from weakly homologous threading templates for these 3 proteins. In Figure 11, the degree of sequence and structure conservation of amino acid residues for these proteins is projected onto the target protein surface and compared to a random distribution. In Figures 12–14, using the target crystal structure, the results of flexible ligand docking by FINDSITE^LHM^ (including refinement) are compared to ligand binding poses predicted by classical docking approaches and the consistently better performance of FINDSITE^LHM^ is demonstrated. In the case of Figure 14 where the RMSDs of LIGIN and random pose prediction are the same as FINDSITE^LHM^, the pyridoxal-5′-phosphate moiety is clearly better placed by FINDSITE^LHM^. All have the same RMSD mainly due to the incorrect placement of the variable region.

**Figure 6 pcbi-1000405-g006:**
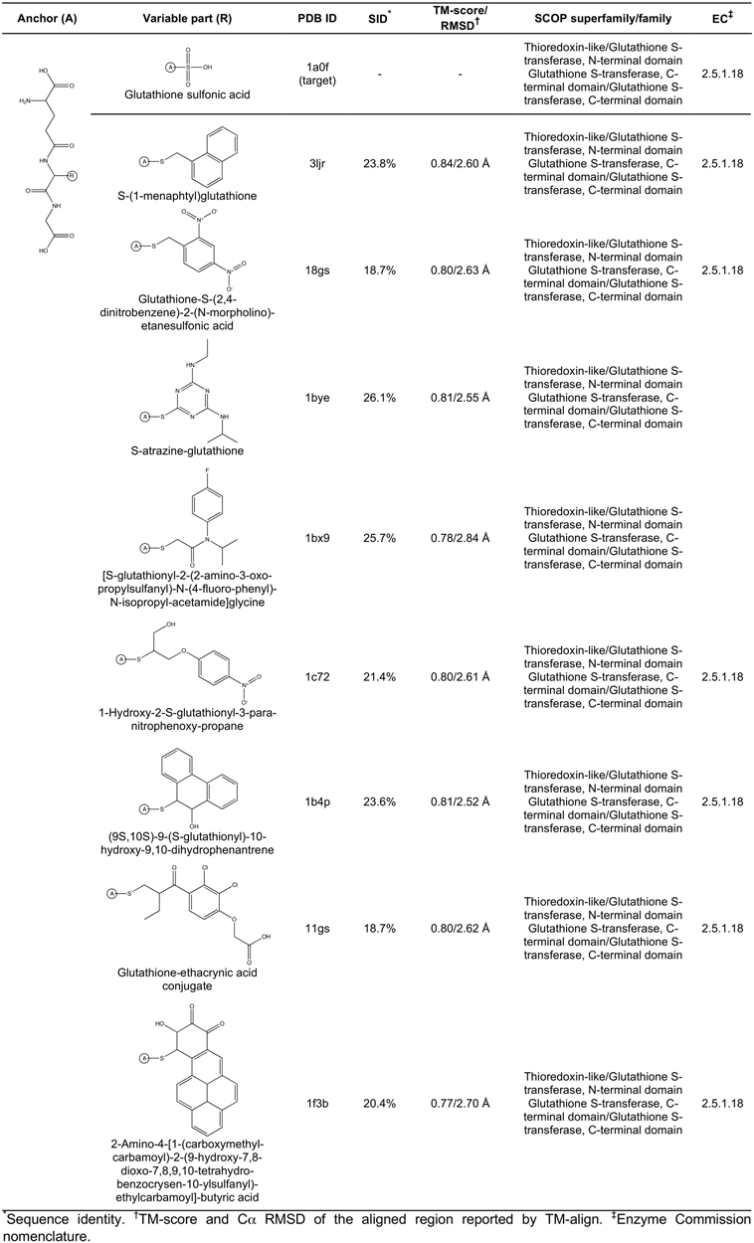
Caption as in Figure 5.

**Figure 7 pcbi-1000405-g007:**
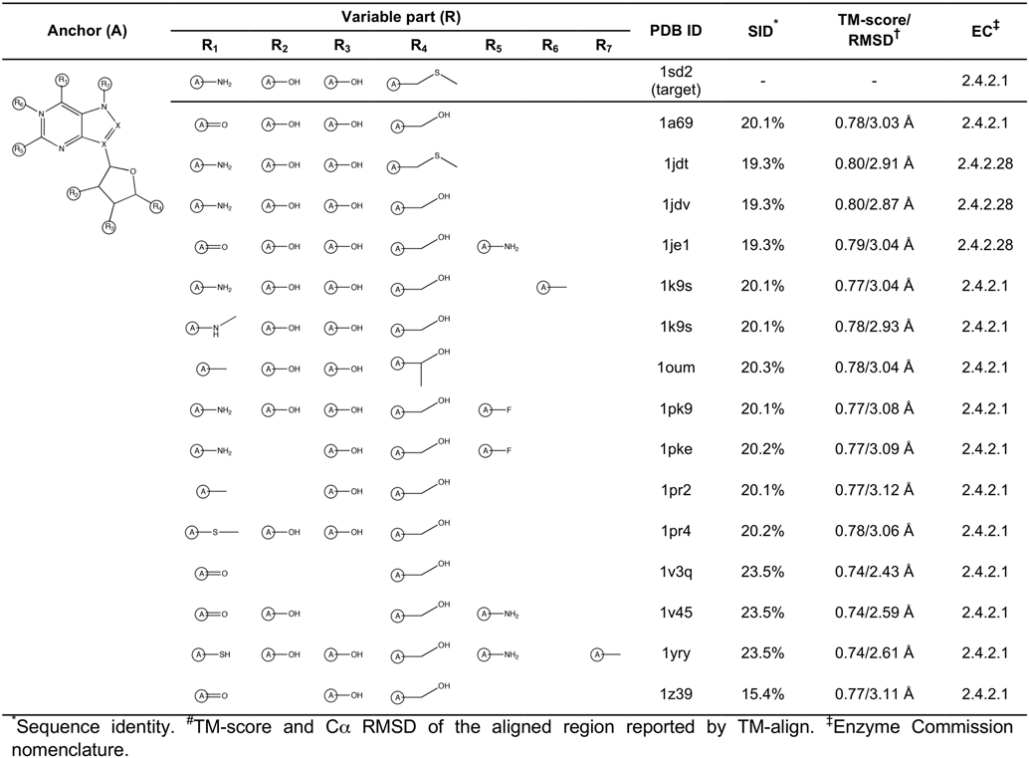
Ligand anchor identification for the human MTA phosphorylase (PDB-ID: 1sd2; SCOP superfamily/family: Purine and uridine phosphorylases/Purine and uridine phosphorylases; EC: 2.4.2.28). Common anchor substructure (A) identified from weakly homologous threading templates as well as different variable groups (at the positions R_1_–R_7_) found in ligands complexed with the template proteins are presented.

**Figure 8 pcbi-1000405-g008:**
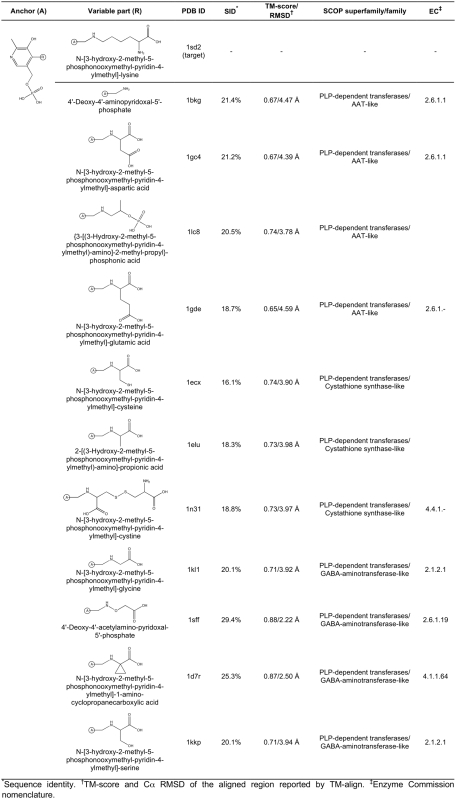
Ligand anchor identification for lysine aminotransferase from *M. tuberculosis* (PDB-ID: 2cjd). Common anchor substructure (A) identified from weakly homologous threading templates as well as different variable groups (R) found in ligands complexed with the template proteins are presented.

**Figure 9 pcbi-1000405-g009:**
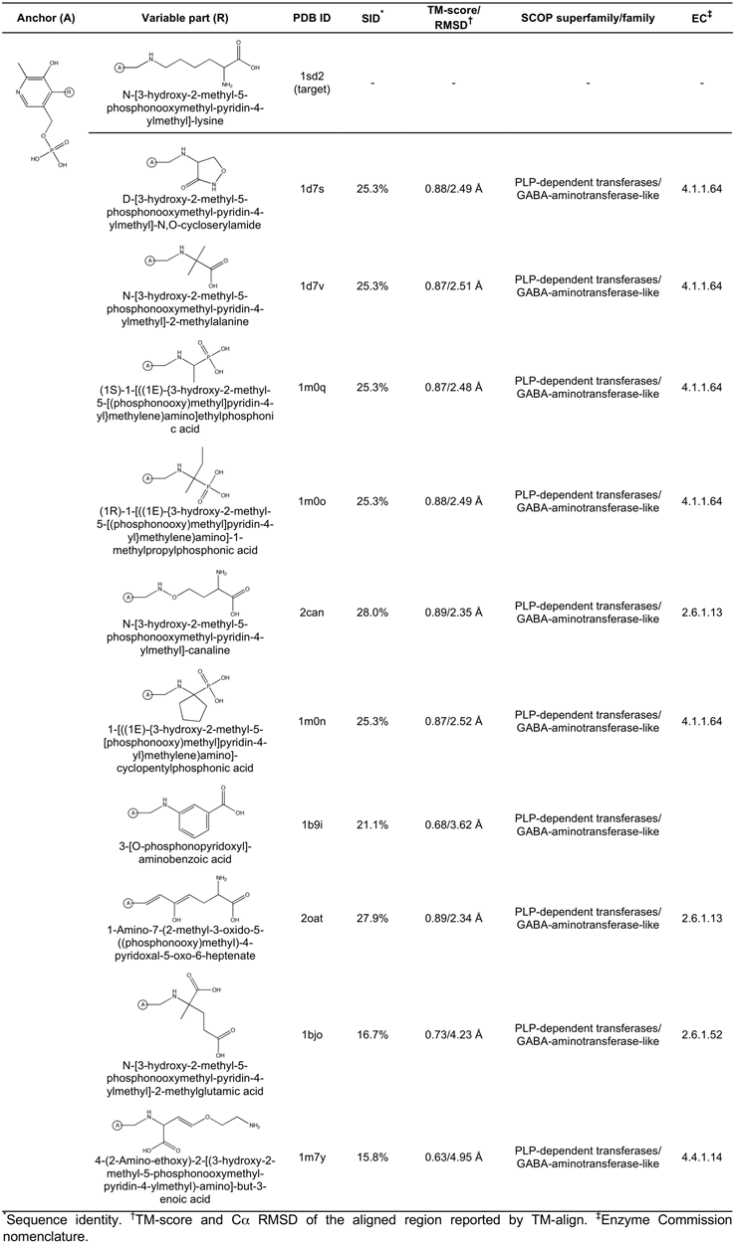
Caption as in Figure 8.

**Figure 10:**
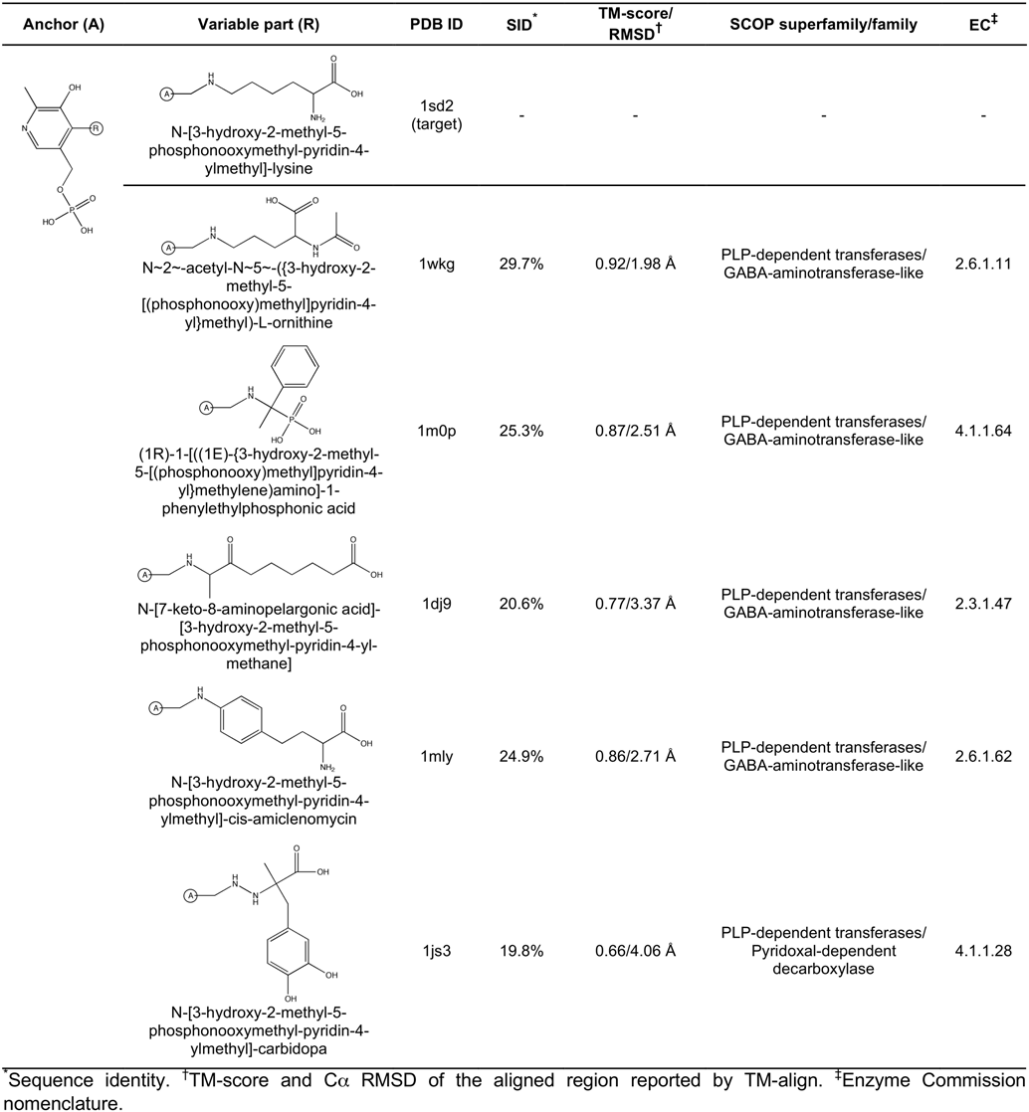
Caption as in Figure 8.

**Figure 11:**
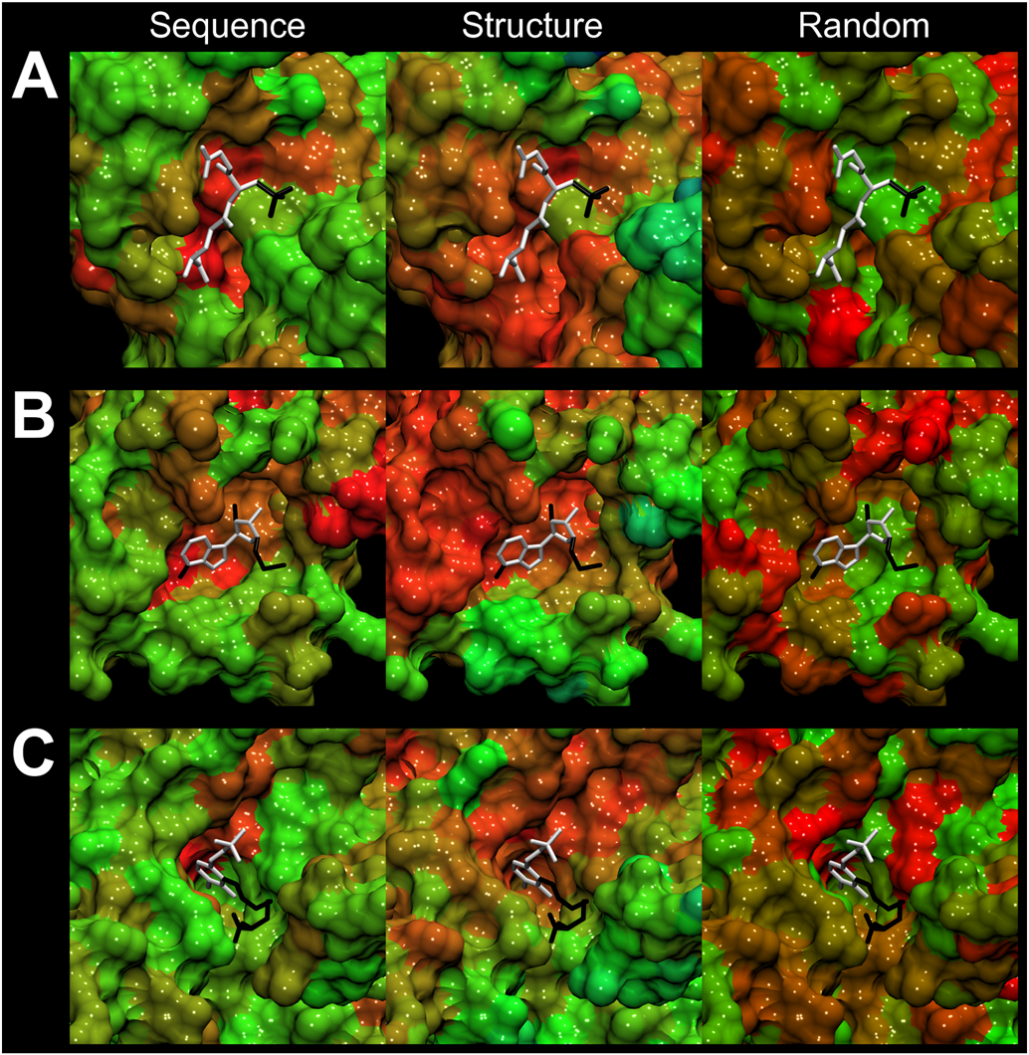
Sequence and structure conservation for the selected ligand-binding sites. (A) Glutathione sulfonic acid complexed with glutathione S-transferase, PDB-ID: 1a0f; (B) 5′-methylthiotubercidin complexed with MTA phosphorylase, PDB-ID: 1sd2; and (C) lysine and piridoxal-5′-phosphate complexed with lysine aminotransferase, PDB-ID: 2cjd. Sequence entropy (red – low, green – high), normalized crystallographic B-factors (red – low, green – high) and random value (red – 0.0, green – 1.0) are presented in left, middle and right column, respectively. The “anchor” part of the molecule is presented in white, whereas the variable part is shown in black.

**Figure 12:**
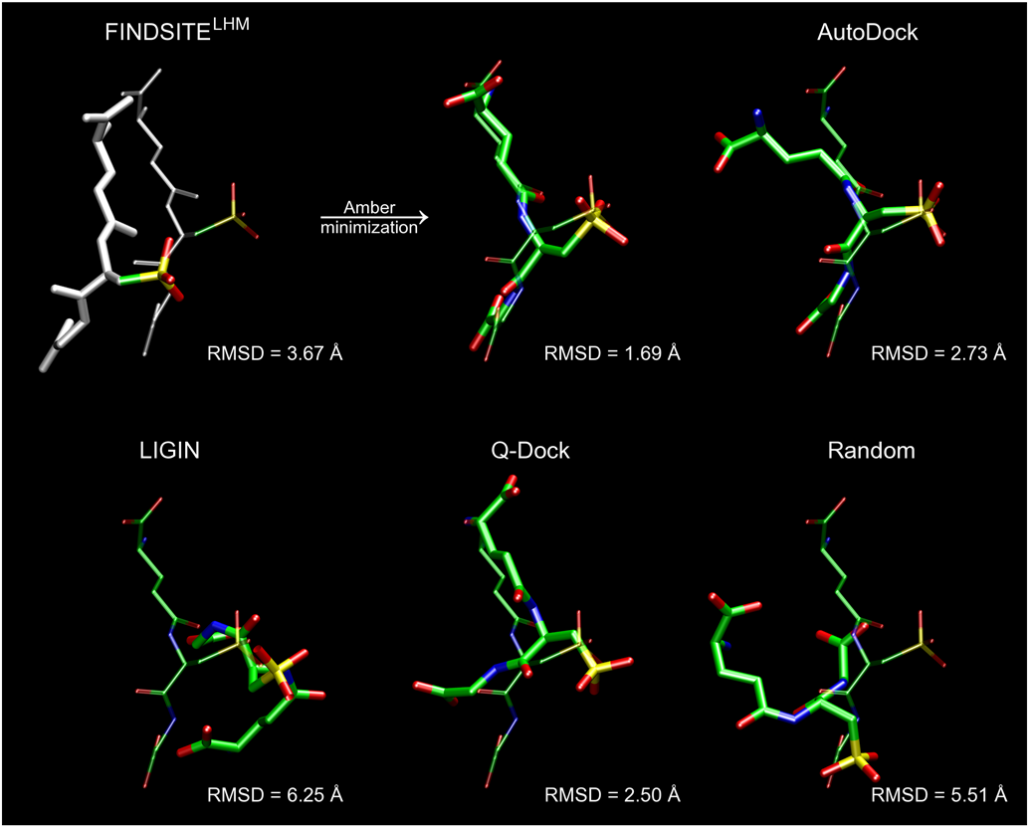
Ligand binding pose prediction for glutathione S-transferase (PDB-ID: 1a0f). Predicted poses (thick sticks) from FINDSITE^LHM^ (superimposed ligand with the anchor portion colored in white and minimized conformation), AutoDock, LIGIN, Q-Dock and a randomly placed ligand are compared to the experimental binding pose (thin sticks). RMSD values were calculated for heavy atoms.

**Figure 13 pcbi-1000405-g013:**
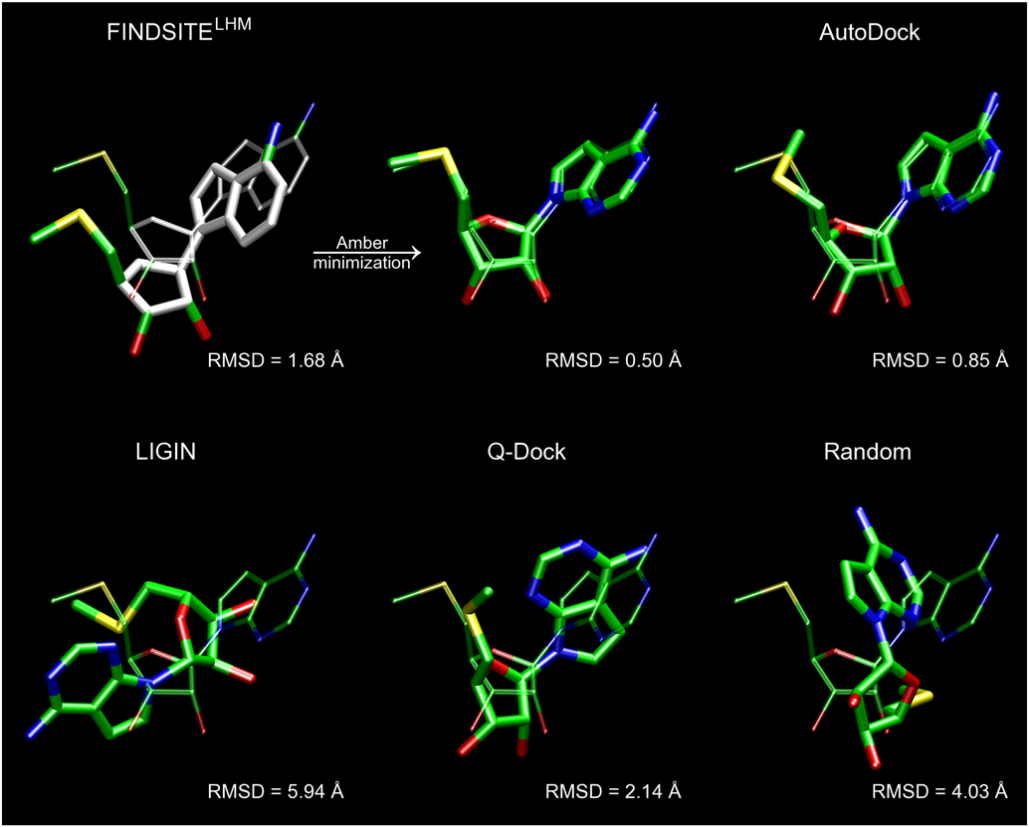
Ligand binding pose prediction for MTA phosphorylase (PDB-ID: 1sd2). Description as in Figure 12.

**Figure 14:**
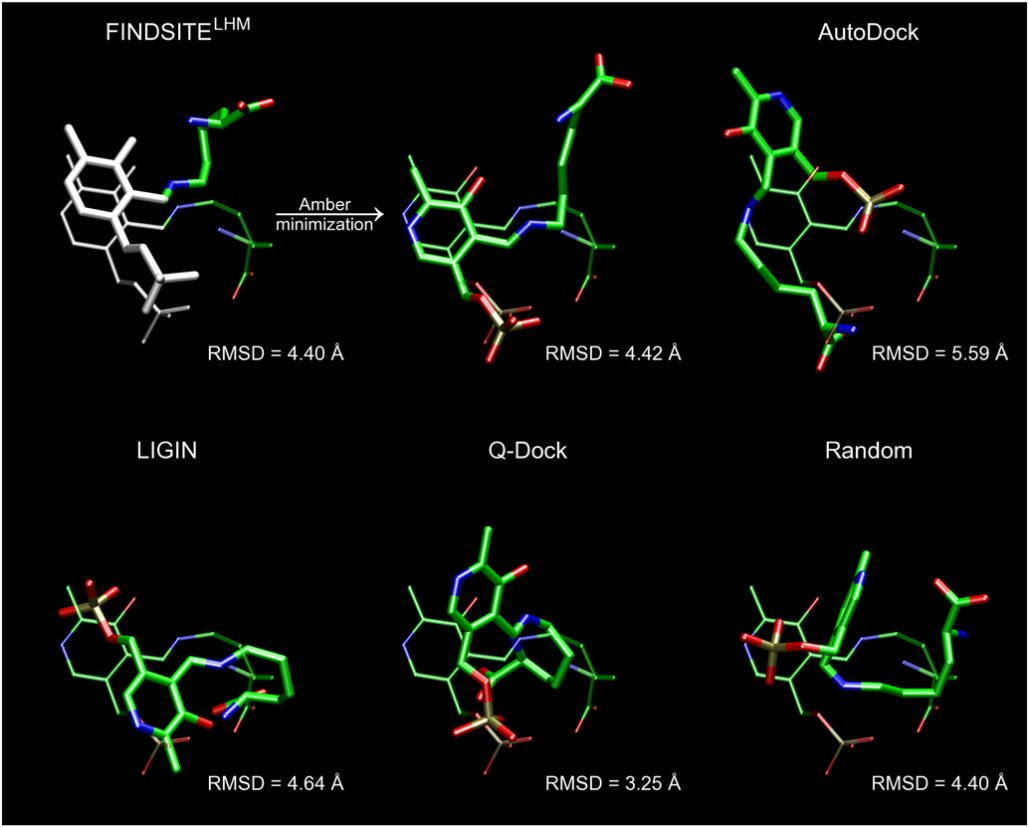
Ligand binding pose prediction for lysine aminotransferase (PDB-ID: 2cjd). Description as in Figure 12.

### Relationship of anchor regions to conserved enzyme substrate substructures

Recently, a detailed picture of the evolution and diversification of enzyme function was drawn from the analysis of conservation of substrate substructures in 42 major enzyme superfamilies [30]. Based on graph isomorphism analysis, highly conserved substructures were identified in all substrates of a particular enzyme superfamily. For the remaining substrate substructures, called reacting substructures, substantial variation in chemical properties within the superfamily was found. Systematic analysis of the substrates in 42 major SCOP [31] enzyme superfamilies revealed chemically conserved patterns that typify individual superfamilies [30]. This approach is very similar in spirit to FINDSITE^LHM^; both demonstrate how evolutionary pressure directs the evolution of protein molecular function. The structural and chemical patterns of enzyme substrates, or small ligands in general, have been conserved during evolution due to the strong conservation of the structural and chemical features of the binding site residues.

**Figure 15:**
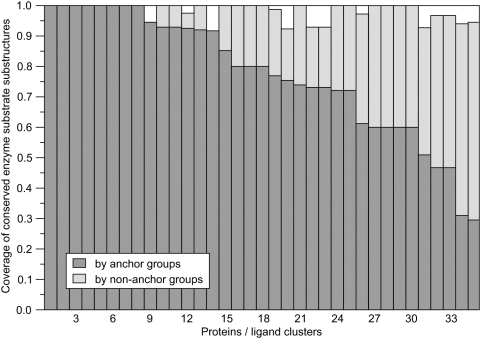
Coverage by anchor and non-anchor functional groups of conserved enzyme substrate substructures from 35 ligand clusters identified for 24 enzymes identified by Babbitt and coworkers [30].

We next analyzed the overlap between the conserved substrate substructures (CSSs) identified at the SCOP superfamily level [30] and the anchor regions in ligands bound to evolutionarily related proteins selected by threading. The results presented in Figure 15 show that the highly conserved substructures of the enzyme substrates identified by Babbitt and colleagues [30] to a large extent overlap with the anchor substructures detected by our threading-based approach; in over 70% of the cases, the anchor substructure covers at least 70% of CSS's atoms. Detailed results obtained for 4-α-glucanotransferase from *T. litoralis* (PDB-ID: 1k1w) and D-xylose isomerase from *Arthrobacter sp.* (PDB-ID: 1die) are presented in Tables S2 and S3, respectively. We find that the highly conserved substructures of the enzyme substrates frequently overlap with the conserved anchor substructures detected by our threading-based approach. The set of ligands that bind to the common binding site in distantly evolutionarily related proteins contain a set of strongly conserved “anchor” functional groups and “variable” regions that account for a specificity toward a particular family member.

As a consequence of the ligand clustering procedure that precedes anchor identification, the anchor substructures typically contain more atoms than CSSs and are not confined to enzymes. Both features are important for practical application in ligand docking by homology modeling, as demonstrated by FINDSITE^LHM^ simulations, where the consensus anchor-binding mode is used as a reference framework for the superposition of a query ligand. Furthermore, common anchor substructures are observed across ligands bound to weakly related proteins that belong to more than one superfamily. These subtle evolutionary relationships detected by sensitive threading techniques [32],[33] are of paramount importance for novel biopharmaceutical discovery that could be accounted for to identify potential off-site drug targets and reduce side effects.

### Application of FINDSITE/FINDSITE^LHM^ to ligand screening

HIV-1 protease plays a crucial role in the life cycle of HIV [34],[35]; thus, it is an important drug target for AIDS treatment with a number HIV-1 protease inhibitors identified [36],[37]. Several (Table 3) are FDA-approved anti-HIV drugs. Here, we selected HIV-1 protease as an example to demonstrate the performance of FINDSITE^LHM^ in ligand-based virtual screening using the coverage of anchor substructures as a simple scoring function.

**Table 3:** Library ranks assigned to FDA-approved drugs in virtual screening for HIV-1 protease inhibitors.

Generic name*	CAS number†	Max TC‡	Library rank§		
			FINDSITE	FINDSITE^LHM^	FINDSITE/FINDSITE^LHM^
Amprenavir	161814-49-9	0.470	13,552	45,271	16,549
Atazanavir	198904-31-3	0.472	**4,766**	**1,661**	***520***
Darunavir	206361-99-1	0.424	30,287	61,485	35,740
Fosamprenavir	226700-81-8	0.434	28,659	***79***	**5,041**
Indinavir	150378-17-9	0.576	***878***	**1,434**	***119***
Lopinavir	192725-17-0	0.660	***32***	**1,836**	***92***
Nelfinavir	159989-64-7	0.595	**5,013**	12,514	**2,227**
Ritonavir	155213-67-5	0.459	28,511	**5,181**	6,481
Saquinavir	127779-20-8	0.596	***87***	**7,397**	***650***
Tipranavir	174484-41-4	0.398	26,044	***22***	**4,244**

*: From: http://www.fda.gov/oashi/aids/virals.html.

**†:** CAS Registry at http://www.cas.org/.

**‡:** Maximal Tanimoto coefficient to template-bound ligands (<35% target -template sequence identity).

**§:** Ranks assigned by FINDSITE, FINDSITE^LHM^ and these resulted from data fusion (FINDSITE/FINDSITE^LHM^); the screening library consists of 124,363 compounds; ranks in bold and italics are within the top 5% and 1% of the library, respectively.

**Figure 16:**
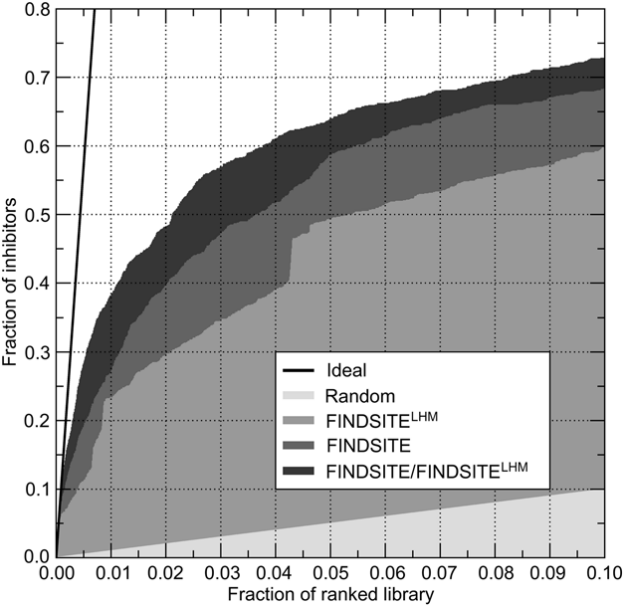
Enrichment behavior for FINDSITE (molecular fingerprints) and FINDSITE^LHM^ (anchor coverage) approaches compared to a random ligand selection in virtual screening for HIV-1 protease inhibitors. FINDSITE/FINDSITE^LHM^ corresponds to the results obtained by applying data fusion.

The performance of FINDSITE^LHM^ alone and in combination with FINDSITE in virtual screening for HIV-1 protease inhibitors is presented in Figure 16. Both FINDSITE and FINDSITE^LHM^ perform considerably better than a random ligand selection. The molecular fingerprints constructed by FINDSITE recovered slightly more known active compounds in the top-ranked fraction of the screening library than anchor-based FINDSITE^LHM^; the enrichment factor calculated for the top 1% (10%) is 27.0 (6.8) and 23.3 (5.9) for FINDSITE and FINDSITE^LHM^, respectively. Clearly, fusion by ranks outperforms the individual scoring functions with the enrichment factor of 38.1 (7.3) for the top 1% (10%) of ranked ligands. These results suggest that the anchor-based approach is able to detect active compounds for which the fingerprint-based method assigns relatively low score. Furthermore, using the combined FINDSITE/FINDSITE^LHM^ approach, 4 (7) out of 10 FDA-approved HIV-1 protease inhibitors are found in the top 1% (5%) of the screening library (Table 3).

## Discussion

Conservation of protein sequence and structural patterns is widely used to study protein molecular function [38]–[40]. Indeed, the structural and chemical characteristics of a binding site are important for understanding ligand selectivity and cross-reactivity [41],[42]. In that regard, our sequence entropy analysis suggests that residues contacting anchor functional groups have been subjected to higher evolutionary conservation pressure than those contacting ligand variable regions. Furthermore, the conservation of the anchor-binding pose is consistent with the relatively low experimental B-factors observed for residues contacting anchor functional groups. The significantly higher structural plasticity of variable region binding residues could reflect the different types/sizes of functional groups found in the ligand variable substructures that might be responsible for ligand specificity for particular protein family members.

Binding site analysis also has practical implications. In the simplest case, using the ligand binding modes extracted from closely related structures and incorporated as spatial restraints in protein structure modeling provides better homology models of protein binding sites [43]. In large-scale computational experiments involving ligand docking, using the AnnoLyze approach the transfer of ligands from known structures of closely related protein-ligand complexes is an attractive alternative to CPU-expensive, classical ligand docking approaches [44]. Here, we have shown that this idea is in fact more general and applies to evolutionarily distant proteins. Indeed, evolution provides a type of signal averaging to identify the essential features associated with ligand binding. This insight can be profitably exploited in a variety of contexts.

For example, for evolutionary distant proteins, we identify the subset of ligands whose pose is conserved, viz. the anchor region. Then, based on the observation that across a set of weakly related proteins, not only is the chemical identity of anchor functional groups strongly conserved but also the anchor binding mode, with an average pairwise RMSD <2.5 Å in most cases. FINDSITE^LHM^ uses the consensus binding mode of an anchor substructure as the reference coordinates to perform rapid flexible ligand docking by superposition. This results in an average ligand heavy atom RMSD from native of 2.5 Å for those ligands that contain a significant portion of the anchor region. Moreover, for predicted protein structures, with considerably less CPU time, FINDSITE^LHM^ outperforms all-atom ligand docking approaches in terms of the fraction of recovered binding residues and specific native contacts.

The accuracy of FINDSITE^LHM^ is affected by several factors: First, for a given target, the set of evolutionarily related template structures needs to be identified. Given the improvements in threading approaches [23],[45] and the completeness of the fold library [46], one can expect to obtain a set of templates for the majority of single domain targets. Next, the docking performance of FINDSITE^LHM^ is well correlated with the overall accuracy of binding pocket prediction by FINDSITE. Typically, high accuracy in ligand binding pose prediction requires the binding site to be precisely detected within a distance of 2 Å. This level of accuracy in pocket prediction is usually achieved for Easy targets, as classified by FINDSITE [21]. Finally, the average accuracy of the binding mode prediction by FINDSITE^LHM^ decreases with the decrease in the coverage of the anchor substructure by the target ligand as well as with the decrease in the degree of the anchor structural conservation. Here, the growing number of protein crystal structures solved in the complexed state with chemically diverse small organic molecules expands the pool of suitable targets for FINDSITE^LHM^. It is noteworthy from the practical point of view that all these properties can be calculated during the modeling procedure, without knowing the native binding pose. Thus the expected accuracy of FINDSITE^LHM^ in ligand binding pose prediction can be estimated with fairly high confidence.

Also as shown for HIV-1 protease, using just the target protein's sequence as input, FINDSITE/FINDSITE^LHM^ can efficiently and rather accurately rank a large ligand library. Since for the majority of gene products, at least weakly homologous proteins can be identified in structural databases by current threading methods [23] and approximately correct protein models can be generated by protein structure prediction techniques [10],[12],[13], FINDSITE^LHM^ offers the possibility of proteome-scale structure-based virtual screening for novel biopharmaceutical discovery. This would have a great advantage over just screening single proteins. It affords the possibility of identifying lead compounds with desired selectivity that could be further exploited at the outset of the drug development process to reduce side effects.

We note that similarity in global fold alone is usually insufficient for effective function interference and results in a high false positive rate [47]. For that reason, the most effective function prediction methods, such as ProFunc [48], AnnoLite [44] or Mark-Us [49] typically combine structure- and sequence-based techniques. In that respect, an important component of FINDSITE/FINDSITE^LHM^ is the template selection by threading that employs a strong sequence profile term [23]. This allows the detection of evolutionarily distant homologues [21] with clear functional relationships to the protein of interest not only in terms of the localization of the binding site, but also in the detailed chemical and structural aspects of ligand binding, particularly those that impart binding specificity. Thus, threading provides a richness to functional annotation that to date was not fully exploited.

## Methods

### Dataset

High quality protein–ligand complex X-ray structures were taken from the Astex diverse set used to validate the GOLD docking algorithm [50] and from a non-redundant Q-Dock dataset [22]. In the Astex set, we excluded complexes in which the binding site is formed by more than one protein chain. From the Q-Dock set, we exclude proteins with >35% sequence identity to any protein in the Astex set. We only include proteins for which at least 5 ligand-bound weakly homologous threading templates can be identified by protein threading and the binding pocket can be predicted by FINDSITE [21] within 4.5 Å from the bound ligand; this represents about 67% of protein targets. The final dataset consisting of 711 complexes is found at http://cssb.biology.gatech.edu/skolnick/files/FINDSITELHM.

In addition to the crystal structures used as the target proteins, we evaluated the performance of FINDSITE^LHM^ in ligand docking against weakly homologous protein models for the Dolores dataset [22],[29] of 205 protein models generated by our protein structure prediction protocol, TASSER [13].

### Binding pocket prediction

For a given amino acid sequence, the PROSPECTOR_3 threading algorithm [23] is used to identify weakly homologous structure templates where templates with >35% sequence identity to target protein are excluded. Structures that bind a ligand are identified by FINDSITE [21] and superimposed onto the reference crystal structure by TM-align [25]. FINDSITE employs an average linkage clustering procedure to cluster the centers of mass of template-bound ligands to detect putative binding sites and then ranks them by the number of ligands.

### Anchor substructure definition

Template-bound ligands that occupy top-ranked predicted binding pockets are clustered using a SIMCOMP similarity (SC) cutoff of 0.7. SIMCOMP is a chemical compound-matching algorithm that provides atom equivalences [26]. Each cluster of ligand molecules is used to detect an anchor substructure. The equivalent atom pairs provided by SIMCOMP are projected onto ligand functional groups. Here, we used the set of 17 functional groups defined in [22]. The anchor substructure is defined as a maximum set of conserved functional groups present in at least 90% of the ligands from a single cluster.

### Protein sequence conservation

The degree of sequence variability was calculated for each consensus binding residue using Shannon's information entropy [51]:
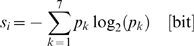
(1)where *p_k_* is the probability that the *i*-th residue position is occupied by an amino acid of class *k*, with the amino acid classification given in [52]. The sequence entropy was calculated only for ligand-bound threading templates that share a common binding pocket. Residue equivalences were provided by TM-align [25].

### Protein structure conservation

Raw experimental B-factors were extracted from the PDB [53] and normalized using the procedure described in [54], with outliers detected and removed using the median-based method [55].

### Ligand docking by FINDSITE^LHM^

The FINDSITE^LHM^ docking procedure superimposes the target ligand onto the consensus binding pose, the anchor conformation averaged over the seed compounds (the largest set of compounds that have their anchor substructures within a 4 Å RMSD from each other), of the identified anchor substructure. *We note that no structural information from the crystal structure of the target complex is used*. If multiple anchor substructures are detected, we select the one derived from the cluster of template-bound ligands with the highest average chemical similarity to the target ligand, as assessed by its SIMCOMP score [26]. This maximizes the coverage of the selected anchor. If atom equivalences for non-anchor atoms can be established between the target ligand and any template-bound ligand, their positions are also included in the set of the reference coordinates. Often, by including additional coordinates, approximately correct positions of ligand variable groups can provide a good initial conformation for post-docking refinement, e.g. in Figures 3, 12, and 13. If none of the identified anchor substructures is covered by the target ligand, it is randomly placed in the predicted pocket. Ligand flexibility is accounted for by the superposition of multiple conformations of the target ligand (for details see classical ligand docking protocols). The conformation that can be superposed onto the reference coordinates with the lowest RMSD to the predicted anchor pose is selected as the final model.

### All-atom refinement

Crude models of protein-ligand complexes generated by FINDSITE^LHM^ were optionally refined by a simple energy minimization in Amber 8 [28]. We used the Amber force field 03 [56] for proteins and the general Amber force field [57], GAFF, for ligands. The parameterization of ligands was done in a fully automated fashion with the aid of Antechamber 1.27 [58]. If necessary, the system was neutralized by calculating a Culombic potential on the grid of 1 Å using LEaP (Amber 8) in order to place chloride (sodium) ions at the positions of the highest (lowest) electrostatic potential around the initial protein-ligand complex. Protein atoms were fixed, while the ligand conformation was energy minimized in vacuum by 1000 cycles of a steepest-descent procedure, followed by 1000 cycles of a conjugate gradient procedure.

### Classical ligand docking

#### AutoDock

We used AutoDock 3 [4] in the flexible ligand docking simulations. Input files for both receptors and ligands were prepared using MGL Tools 1.5.2 [59]. A grid spacing of 0.375 Å was used, with the box dimensions depending on the target ligand size, such that the ligand's geometric center was not allowed to move more than 7 Å away from the predicted binding pocket center. Each docking simulation consisted of 100 runs of a genetic algorithm (GA) using the default GA parameters. The lowest-energy conformation was taken as the final docking result.

#### Q-Dock

We followed the protocol for low-resolution ligand docking using Replica Exchange Monte Carlo (MC) described in detail in [22]. Ligand flexibility was accounted for by docking the ensemble of, at most 50, non-redundant (1 Å pairwise RMSD cutoff) discrete ligand conformations; the number of conformations depends on the number of rotatable bonds and the hybridization of bonded atoms. We used a 7 Å radius docking sphere (7 Å is the maximal allowed distance between the ligand's geometric center and the center of the predicted binding pocket). The simulations utilized 16 replicas and consisted of 100 attempts at replica exchange and 100 MC steeps between replica swaps. The final model corresponds to the lowest-energy conformation.

#### LIGIN

This all-atom docking approach uses molecular shape complementarity and atomic chemical properties to predict the optimal binding pose of a ligand inside the receptor binding pocket [24]. LIGIN is a rigid-body docking approach that by default ignores ligand flexibility. Here, we adopted the idea of ligand docking using conformational ensembles [22],[60],[61] to mimic the ligand flexibility in LIGIN. To the best of our best knowledge, such pseudo-flexibility in LIGIN was never before tested. For a given target, we used exactly the same ensemble of multiple ligand conformations as in Q-Dock simulations and FINDSITE^LHM^, and docked each of them into the predicted binding site using LIGIN. The docking procedure was repeated 1000 times for each ligand conformer. The final binding mode corresponds to that of maximal complementarity found in the complete set of ligand conformers. Atom types were assigned using LPC [62]; no receptor residues were permitted to have steric overlap with the ligand.

### Highly conserved substructures observed in ligands complexed to evolutionarily related proteins

From our dataset of 711 protein-ligand complexes, we selected only enzymes in which the anchor substructure (or multiple anchor substructures) derived for the top-ranked predicted binding pockets consists of ≥50% and ≤90% of the average ligand molecule's size and matches the native ligand. Subsequently, native ligands were scanned for the presence of CSSs. Here, we used the collection of the CSSs compiled for 42 major enzyme superfamilies by Babbitt and colleagues [30], from which we removed those substructures that consist of less than 5 atoms. A CSS was considered to be present in the native ligand if the native ligand atoms cover at least 90% of its atoms, as reported by SIMCOMP [26]. This procedure resulted in 24 enzymes and 35 ligand clusters. Next, for each cluster and the associated anchor substructure, we examined the fraction of CSS's atoms covered by the anchor functional groups as well as the fraction covered by the non-anchor groups.

### Virtual screening of HIV-1protease

The screening library consists of 1089 known HIV-1 protease inhibitors (MDL activity index: 71523) extracted from the MDL Drug Data Report [63] and 123,274 lead-like background compounds from the Asinex Platinum Collection [64].

A weakly homologous model of HIV-1 protease was generated from the amino acid sequence (PDB: 1w5y) using TASSER [13]. Only distantly related (<35% sequence identity to HIV-1 protease) structure templates were used. The predicted model used in this study has a 4.91 Å (4.09 Å) RMSD to native calculated for all heavy atoms (Cα atoms).

### Scoring functions for virtual screening

We applied two ligand-based virtual screening techniques to rank the screening library: a fingerprint-based method implemented in FINDSITE and simple scoring by the anchor substructure coverage, where the anchor substructures were identified by FINDSITE^LHM^. In both cases, we used a collection of ligands bound to weakly homologous (<35% sequence identity to the target) threading templates identified by PROSPECTOR_3 with a Z-score ≥4. FINDSITE constructs ligand templates for fingerprint-based virtual screening by clustering the molecules that occupy the top-ranked predicted binding site using the Tanimoto coefficient (*TC*) [65] cutoff of 0.7 [21]. Here, we employed the 1,024-bit molecular fingerprints from Daylight Chemical Information Systems [66]. The representative molecules selected from the clusters were used to rank a compound library using a weighted Tanimoto coefficient (*mTC^ave^*):
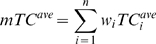
(2)where *n* is the number of ligand clusters, *w_i_* is the fraction of ligands that belong to cluster *i*, and 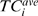 is the averaged *TC* (*TC^ave^*) calculated for the representative ligand from cluster *i* and a library compound.

The overlap between two fingerprints was measured by *TC^ave^* [67]–[69]:
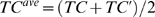
(3)where *TC′* is the *TC* calculated for bit positions set to zero rather than to one as in the traditional *TC* [65].

In virtual screening by anchor coverage, we used the anchor substructures detected for HIV-1 protease by FINDSITE^LHM^ as described in Methods. For a given library compound, we calculated the coverage of the anchor substructure that was derived from the cluster of template-bound ligands with the highest average chemical similarity, as assessed by SIMCOMP score [26]. The screening library was then ranked by decreasing anchor coverage.

Finally, we applied data fusion to combine the results from virtual screening using the fingerprint-based (FINDSITE) and the anchor-based (FINDSITE^LHM^) approaches. Data fusion techniques are commonly used in chemoinformatics to merge screening results generated by different descriptors or scoring functions [70]–[74]. Typically, chemical data fusion employs the combination of rankings from individual screening experiments using one of several different fusion rules, such as MIN, MAX or SUM [75]. Here, we applied the SUM rule that is expected to be less sensitive to noisy input than both extreme rules [70] and is generally preferred when fusion is by rank [71]. For a given library compound *k*, a combined score (*CS*) is calculated from:
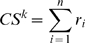
(4)where *n* is the number of ranked lists (in our case, *n* = 2: FINDSITE and FINDSITE^LHM^) and *r_i_* denotes the rank position of the library compound *k* in the *i*-th ranked list.

### Enrichment factor

To assess the performance of FINDSITE/FINDSITE^LHM^ in virtual screening for HIV-1 protease inhibitors, we calculated the enrichment factor (*EF*) [76],[77] for the top 1% and 10% of the ranked screening library:
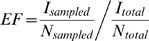
(5)where *I_sampled_* is the number of known HIV-1 protease inhibitors in the top-ranked fraction of *N_sampled_* compounds, *I_total_* and *N_total_* is the total number of inhibitors and the library compounds, respectively.

The maximal enrichment factors for the top 1% and 10% of the ranked library are 100 and 10, respectively. In addition to the enrichment factor, we assessed the results in terms of the enrichment behavior, i.e. the fraction of known inhibitors retrieved in the top-ranked fraction of the ranked screening library.

## Supporting Information

Table S1 Docking times for the Dolores dataset. All docking simulations were performed using a 2.0 GHz AMD Opteron processor. Timings reported for LIGIN, Q-Dock and FINDSITE^LHM^ include the pre-docking generation of ligand conformational ensemble (median: 23 s on a 3.4 GHz P4).
(0.04 MB PDF)

Table S2 Multiple common anchor substructures (blue) identified from weakly homologous threading templates for 4-α-glucanotransferase from *T. litoralis* (PDB-ID: 1k1w) compared to the conserved substrate substructure reported by Chiang *et al.* 2008 (red). The overlap between both substructures is colored in green. The anchor substructures are presented for selected ligand clusters obtained for top-ranked binding pockets.
(0.35 MB PDF)

Table S3 Multiple common anchor substructures (blue) identified from weakly homologous threading templates for D-xylose isomerase from *Arthrobacter sp.* (PDB-ID: 1die) compared to the conserved substrate substructure reported by Chiang *et al.* (red). The overlap between both substructures is colored in green. The anchor substructures are presented for selected ligand clusters obtained for top-ranked binding pockets.
(0.30 MB PDF)
