# Supplementary material for: FINDSITELHM: A Threading-Based Approach to Ligand Homology Modeling
Source: PLoS Comput Biol. 2009 Jun 5;5(6):e1000405. doi: 10.1371/journal.pcbi.1000405 (PMC2685473; doi:10.1371/journal.pcbi.1000405)
Supplement: Table S1 — Docking times for the Dolores dataset. All docking simulations were performed using a 2.0 GHz AMD Opteron processor. Timings reported for LIGIN, Q-Dock and FINDSITELHM include the pre-docking generation of ligand conformational ensemble (median: 23 s on a 3.4 GHz P4). (0.04 MB PDF) [file pcbi.1000405.s001.pdf]

Table S1. Docking times for the Dolores dataset. All docking simulations were performed using a 2.0GHz AMD Opteron processor. Timings reported for LIGIN, Q-Dock and FINDSITE^{LHM} include the pre-docking generation of ligand conformational ensemble (median: 23s on a 3.4GHz P4).

Docking algorithm	Docking time in seconds*
AutoDock†	11158 (286:1)
LIGIN‡	1623 (42:1)
Q-Dock§	1479 (38:1)
FINDSITE^{LHM}, ¶	39 (1:1)
FINDSITE^{LHM} + minimization‖	48 (1.2:1)

*Median values are reported; the numbers in parentheses show the ratio of the docking time to FINDSITE^{LHM}. †100 runs of a genetic algorithm using a grid spacing of 0.375 Å. ‡1000 rounds of optimization for each ligand conformer. §16 replicas, 100 attempts at replica exchange and 100 MC steps between replica swaps. ¶Superposition of each ligand conformer onto the consensus anchor-binding mode. ‖Including all-atom minimization with Amber.
