# Supplementary material for: FINDSITELHM: A Threading-Based Approach to Ligand Homology Modeling
Source: PLoS Comput Biol. 2009 Jun 5;5(6):e1000405. doi: 10.1371/journal.pcbi.1000405 (PMC2685473; doi:10.1371/journal.pcbi.1000405)
Supplement: Table S2 — Multiple common anchor substructures (blue) identified from weakly homologous threading templates for 4-α-glucanotransferase from T. litoralis (PDB-ID: 1k1w) compared to the conserved substrate substructure reported by Chiang et al. 2008 (red). The overlap between both substructures is colored in green. The anchor substructures are presented for selected ligand clusters obtained for top-ranked binding pockets. (0.35 MB PDF) [file pcbi.1000405.s002.pdf]

Table S2. Multiple common anchor substructures (blue) identified from weakly homologous threading templates for 4-α-glucanotransferase from *T. litoralis* (PDB-ID: 1k1w) compared to the conserved substrate substructure reported by Chiang *et al*. 2008 (red). The overlap between both substructures is colored in green. The anchor substructures are presented for selected ligand clusters obtained for top-ranked binding pockets.

	**Ligands**	**PDB-ID**	**SID[*]**	**TM-score/RMSD[†]**	**SCOP superfamily/family**	**EC[‡]**
Target protein	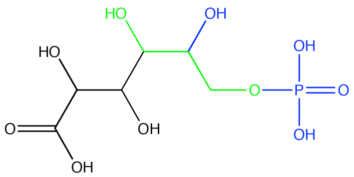 L-guluronic acid 6-phosphate	1k1w	-	-	Ribulose-phosphate binding barrel/Decarboxylase	4.1.2.-
Cluster: 1<br>Templates: 45<br>RMSD[§]: 2.90 Å	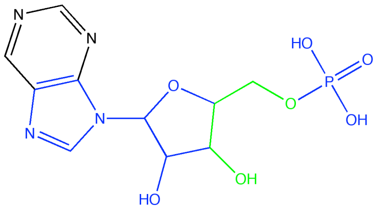 Purine-riboside-5'-monophosphate	1b3o	19.3%	0.54/3.18 Å	Inosine monophosphate dehydrogenase (IMPDH)/ Inosine monophosphate dehydrogenase (IMPDH)	1.1.1.205
	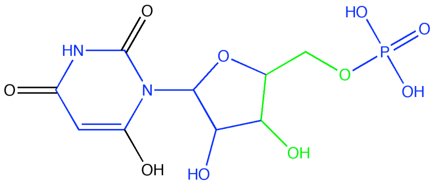 6-Hydroxyuridine-5'-phosphate	1dqx	19.6%	0.62/2.50 Å	Ribulose-phosphate binding barrel/Decarboxylase	4.1.1.23

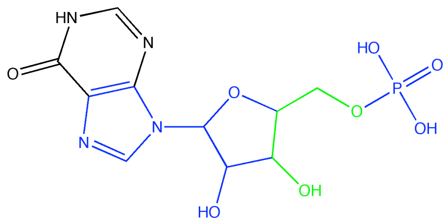<br>Inosinic acid	1jr1	15.8%	0.41/3.50 Å	Inosine monophosphate dehydrogenase (IMPDH)/ Inosine monophosphate dehydrogenase (IMPDH)	1.1.1.20<br>5
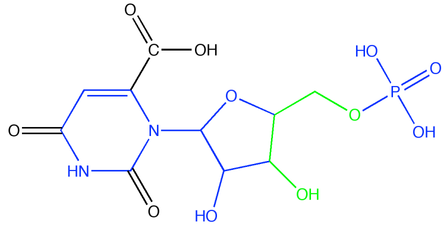<br>Orotidine-5’-monophosphate	1km6	24.8%	0.82/2.12 Å	Ribulose-phosphate binding barrel/Decarboxylase	4.1.1.23
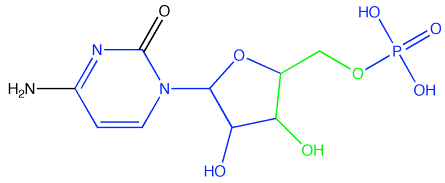<br>Cytidine-5’-monophosphate	1lp6	23.3%	0.82/1.94 Å	Ribulose-phosphate binding barrel/Decarboxylase	4.1.1.23
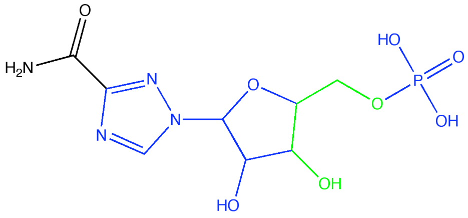<br>Ribavirin monophosphate	1me7	15.1%	0.47/3.56 Å	Inosine monophosphate dehydrogenase (IMPDH)/ Inosine monophosphate dehydrogenase (IMPDH)	1.1.1.20<br>5

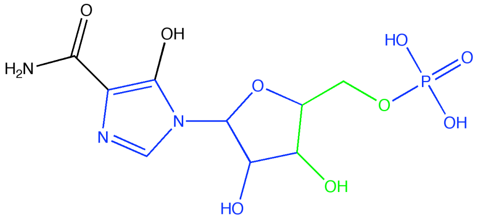 Imidazolium-5-olate-5'-phosphate-2-amino-2-hydroxymethyl-propane-1,3-diol	1mwf	15.2%	0.47/3.55 Å	Inosine monophosphate dehydrogenase (IMPDH)/ Inosine monophosphate dehydrogenase (IMPDH)	1.1.1.205
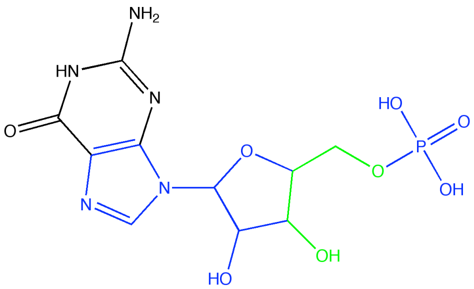 Guanosine-5'-monophosphate	2ble	17.0%	0.49/3.51 Å	-	1.7.1.7
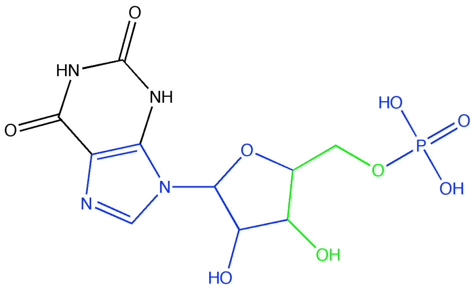 Xanthosine-5'-monophosphate	2czf	25.1%	0.82/2.20 Å	Ribulose-phosphate binding barrel/Decarboxylase	4.1.1.23

	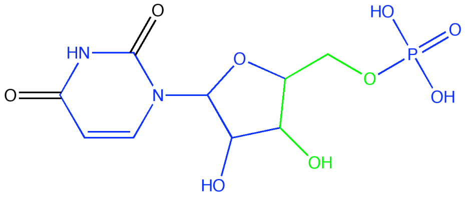<br>Uridine-5'-monophosphate	2ffc	18.1%	0.56/2.65 Å	Ribulose-phosphate binding barrel/Decarboxylase	4.1.3.23
Cluster: 2<br>Templates: 20<br>RMSD§: 3.45 Å	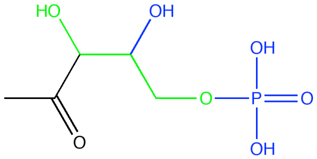<br>1-Deoxy-D-xylulose-5-phosphate	1ixn	20.4%	0.71/2.91 Å	Pyridoxine 5'-phosphate synthase/Pyridoxine 5'-phosphate synthase	-
	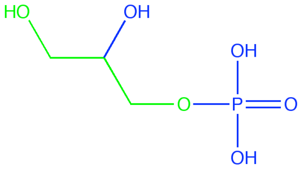<br>Glycerol-3-phosphate	1ixo	20.5%	0.73/2.92 Å	Pyridoxine 5'-phosphate synthase/Pyridoxine 5'-phosphate synthase	-
	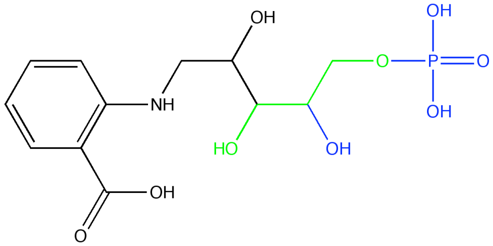<br>1-(O-carboxy-phenylamino)-1-deoxy-D-ribulose-5-phosphate	1lbm	15.5%	0.77/2.96 Å	Ribulose-phosphate binding barrel/Tryptophan biosynthesis enzymes	5.3.1.24

	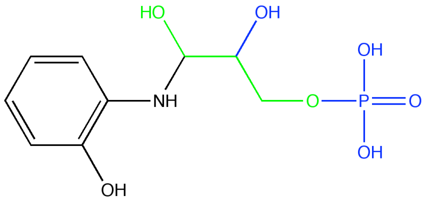 1-[(2-Hydroxylphenyl)-amino]-3-glycerolphosphate	1tjp	16.8%	0.62/3.49 Å	Ribulose-phosphate binding barrel/Tryptophan biosynthesis enzymes	4.2.1.20
	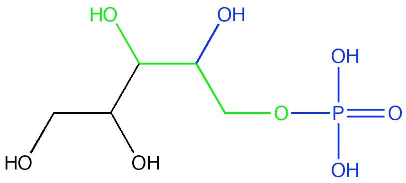 D-xylitol-5-phosphate	2fli	17.7%	0.79/2.95 Å	Ribulose-phosphate binding barrel/D-ribulose-5-phosphate 3-epimerase	5.1.3.-
Cluster: 3<br>Templates: 40<br>RMSD§: 3.83 Å	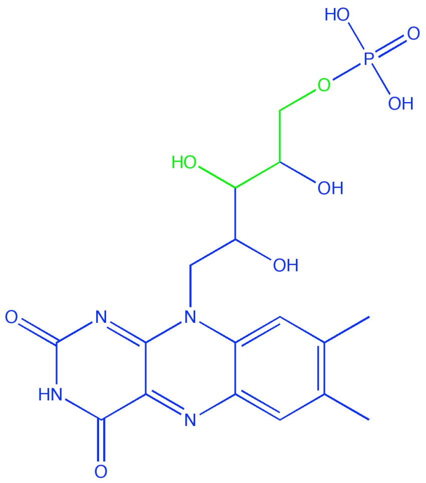 Flavin mononucleotide	1dor	17.5%	0.54/3.28 Å	FMN-linked oxidoreductases/ FMN-linked oxidoreductases	1.3.3.1

[*]Sequence identity. [†]TM-score and Cα RMSD of the aligned region reported by TM-align. [‡]Enzyme Commission nomenclature. [§]Average pairwise RMSD of the anchor heavy atoms calculated for all ligands that belong to a particular cluster.
