# Supplementary material for: FINDSITELHM: A Threading-Based Approach to Ligand Homology Modeling
Source: PLoS Comput Biol. 2009 Jun 5;5(6):e1000405. doi: 10.1371/journal.pcbi.1000405 (PMC2685473; doi:10.1371/journal.pcbi.1000405)
Supplement: Table S3 — Multiple common anchor substructures (blue) identified from weakly homologous threading templates for D-xylose isomerase from Arthrobacter sp. (PDB-ID: 1die) compared to the conserved substrate substructure reported by Chiang et al. (red). The overlap between both substructures is colored in green. The anchor substructures are presented for selected ligand clusters obtained for top-ranked binding pockets. (0.30 MB PDF) [file pcbi.1000405.s003.pdf]

Table S3. Multiple common anchor substructures (blue) identified from weakly homologous threading templates for D-xylose isomerase from *Arthrobacter sp.* (PDB-ID: 1die) compared to the conserved substrate substructure reported by Chiang *et al*. (red). The overlap between both substructures is colored in green. The anchor substructures are presented for selected ligand clusters obtained for top-ranked binding pockets.

	Ligands	PDB-ID	SID*	TM-score/RMSD†	SCOP superfamily/family	EC‡
Target protein	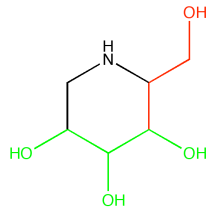 1-Deoxynojirmycin	1die	-	-	Xylose isomerase-like/ Xylose isomerase	5.3.1.5
Cluster: 1<br>Templates: 12<br>RMSD§: 3.49 Å	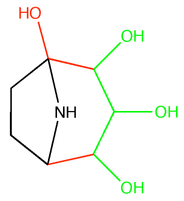 Calystegine B2	2cbv	19.5%	0.53/4.65 Å	(Trans)glycosidases/ Family 1 of glycosyl hydrolase	3.2.1.21

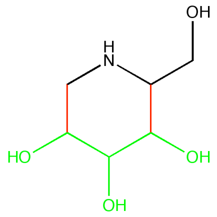 1-Deoxynojirimycin	1oim	19.5%	0.53/4.64 Å	(Trans)glycosidases/ Family 1 of glycosyl hydrolase	3.2.1.21
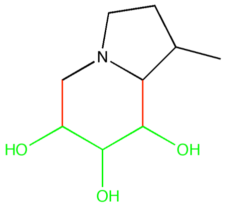 Castanospermine	2cbu	19.7%	0.54/4.67 Å	(Trans)glycosidases/ Family 1 of glycosyl hydrolase	3.2.1.21
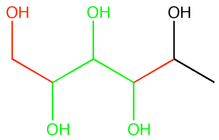 L-rhamnitol	1de5	19.3%	0.68/3.45 Å	Xylose isomerase-like/ L-rhamnose isomerase	5.3.1.14
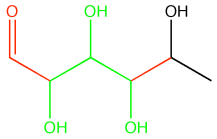 L-rhamnose	1de6	19.3%	0.68/3.50 Å	Xylose isomerase-like/ L-rhamnose isomerase	5.3.1.14

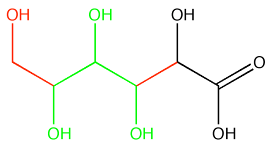 Gluconic acid	1bgg	18.8%	0.53/4.66 Å	(Trans)glycosidases/ Family 1 of glycosyl hydrolase	3.2.1.21
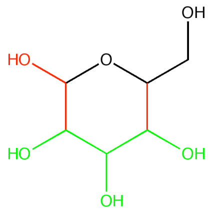 α-D-glucose	1hiz	16.4%	0.55/5.05 Å	(Trans)glycosidases/ Beta-glycanases	3.2.1.8
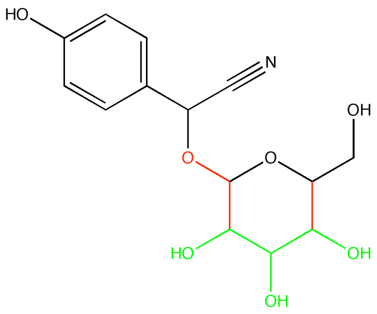 Dhurrin	1e55	20.1%	0.49/4.69 Å	(Trans)glycosidases/ Family 1 of glycosyl hydrolase	3.2.1.21

Cluster: 2<br>Templates: 12<br>RMSD§: 5.61 Å	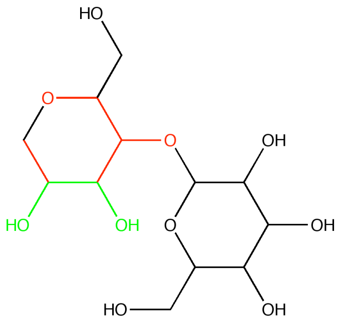<br>β-D-mannose-α-D-mannose	2man	15.3%	0.63/4.38 Å	(Trans)glycosidases/<br>Beta-glycanases	3.2.1.78
	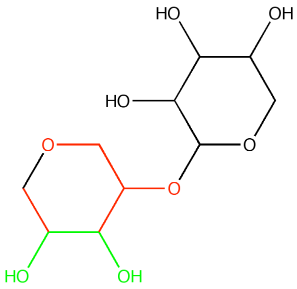<br>Xylobiose	1vbr	17.9%	0.62/4.89 Å	(Trans)glycosidases/<br>Beta-glycanases	3.2.1.8
	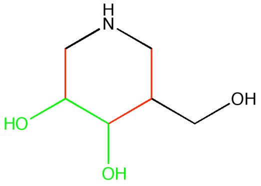<br>5-Hydroxymethyl-3,4-dihydroxypiperidine	1oif	19.8%	0.53/4.61 Å	(Trans)glycosidases/<br>Family 1 of glycosyl hydrolase	3.2.1.21

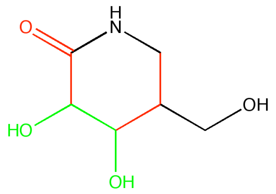<br>(3S,4R,5R)-3,4-dihydroxy-5-(hydroxymethyl)piperidin-2-one	1uz4	19.2%	0.54/4.75 Å	(Trans)glycosidases/ Beta-glycanases	-
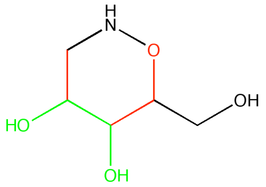<br>Tetrahydrooxazine	1w3j	19.3%	0.53/4.62 Å	(Trans)glycosidases/ Beta-glycanases	3.2.1.21
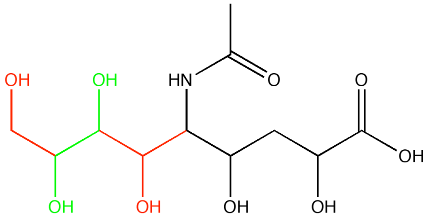<br>2,4,6,7,8,9-Hexahydroxy-5-methylcarboxamido nonanoic acid	1f73	17.5%	0.59/3.92 Å	Aldolase/Class I aldolase	4.1.3.3

	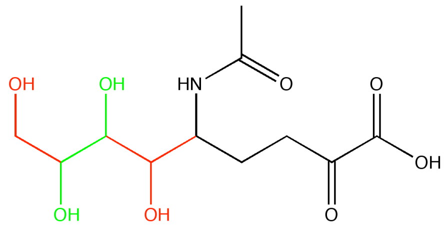<br>6,7,8,9-Tetrahydroxy-5-methylcarboxamido-2-oxononanoic acid	1f74	17.5%	0.58/4.11 Å	Aldolase/Class I aldolase	4.1.3.3
Cluster: 3<br>Templates: 12<br>RMSD§: 3.86 Å	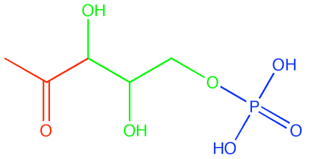<br>1-Deoxy-D-xylulose-5-phosphate	1ixn	19.6%	0.69/3.76 Å	Pyridoxine 5'-phosphate synthase/ Pyridoxine 5'-phosphate synthase	-
	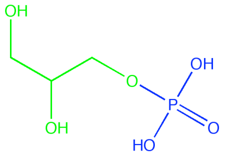<br>Glycerol-3-phosphate	1ixo	19.3%	0.71/3.78 Å	Pyridoxine 5'-phosphate synthase/ Pyridoxine 5'-phosphate synthase	-

	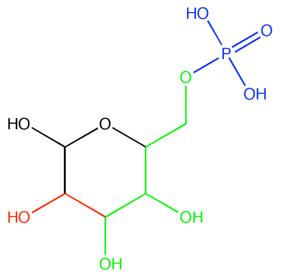 β-Galactose-6-phosphate	4pbg	21.2%	0.51/4.49 Å	(Trans)glycosidases/ Family 1 of glycosyl hydrolase	3.2.1.85
Cluster: 4<br>Templates: 2<br>RMSD§: 0.71 Å	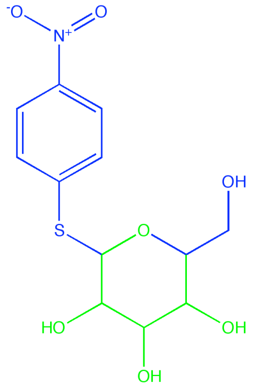 Para-nitrophenyl-1-thio-β-D-glucopyranoside	1e1f	19.5%	0.49/4.67 Å	(Trans)glycosidases/ Family 1 of glycosyl hydrolase	3.2.1.21

*Sequence identity. †TM-score and Cα RMSD of the aligned region reported by TM-align. ‡Enzyme Commission nomenclature. §Average pairwise RMSD of the anchor heavy atoms calculated for all ligands that belong to a particular cluster.
